# Mapping mitochondrial morphology and function: COX-SBFSEM reveals patterns in mitochondrial disease

**DOI:** 10.1038/s42003-024-07389-7

**Published:** 2025-01-09

**Authors:** Julie Faitg, Tracey Davey, Ross Laws, Conor Lawless, Helen Tuppen, Eric Fitton, Doug Turnbull, Amy E. Vincent

**Affiliations:** 1 Wellcome Centre for Mitochondrial Research, Translational and Clinical Research, Faculty of Medical Sciences, Newcastle University, Newcastle, UK; 2 Electron Microscopy Research Services, Newcastle University, Newcastle, UK; 3 NIHR Biomedical Research Centre Research Centre, Translational and Clinical Research, Faculty of Medical Sciences Newcastle University, Newcastle, UK; 4 John Walton Muscular Dystrophy Research Centre, Translational and Clinical Research, Faculty of Medical Sciences Newcastle University, Newcastle, UK

**Keywords:** Mechanisms of disease, Histocytochemistry

## Abstract

Mitochondria play a crucial role in maintaining cellular health. It is interesting that the shape of mitochondria can vary depending on the type of cell, mitochondrial function, and other cellular conditions. However, there are limited studies that link functional assessment with mitochondrial morphology evaluation at high magnification, even fewer that do so in situ and none in human muscle biopsies. Therefore, we have developed a method which combines functional assessment of mitochondria through Cytochrome c Oxidase (COX) histochemistry, with a 3D electron microscopy (EM) technique, serial block-face scanning electron microscopy (SBFSEM). Here we apply COX-SBFSEM to muscle samples from patients with single, large-scale mtDNA deletions, a cause of mitochondrial disease. These deletions cause oxidative phosphorylation deficiency, which can be observed through changes in COX activity. One of the main advantages of combining 3D-EM with the COX reaction is the ability to look at how per-mitochondrion oxidative phosphorylation status is spatially distributed within muscle fibres. Here we show a robust spatial pattern in COX-positive and intermediate-fibres and that the spatial pattern is less clear in COX-deficient fibres.

## Abstract (web summary)

Innovative COX-SBFSEM method integrates mitochondrial function and morphology assessment to study muscle biopsies from patients with mtDNA deletions, revealing spatial patterns in oxidative phosphorylation within muscle fibers.

## Introduction

In skeletal muscles, there are two mitochondrial populations, subsarcolemmal mitochondria (SS), located beneath the cell membrane, and intermyofibrillar mitochondria (IMF), located between myofibrils^1^. SS mitochondria are generally more spherical, with some exceptions of elongated mitochondria which protrude into the intermyofibrillar space^2,3^. IMF mitochondria, on the other hand, appear to be organised into an interconnected reticulum, with more complex mitochondria^2,4^. It is also worth noting that, as well as morphological differences, these two populations exhibit functional specialisation including differences in capacity to generate ATP^5^. Importantly, despite their morphological and functional distinctions, these two mitochondrial pools are physically interconnected and not completely separate entities^2,6^, forming a dynamic and integrated mitochondrial network within muscle fibres.

Mitochondrial DNA (mtDNA) mutations are associated with mitochondrial oxidative phosphorylation (OXPHOS) dysfunction in skeletal muscle. These mtDNA mutations can be inherited, occur sporadically early in embryogenesis or arise as a result of nuclear gene mutations that impact mtDNA maintenance. Since mtDNA is present in hundreds to thousands of copies per cell, a mutation in one or a small number of mtDNA copies has negligible impact. However, when present in higher proportions of the mtDNA within a single cell, a mtDNA mutation may exceed a biochemical threshold^7^ and cause mitochondrial dysfunction. MtDNA encodes genes for subunits of complex I (CI), complex III (CIII), complex IV (CIV) and complex V (CV), as well as tRNAs and rRNAs for mitochondrial translation. As such, mtDNA mutations may give rise to deficiency in CI, III, IV or V depending on which genes are impacted, with cytochrome c oxidase (COX) or CIV being the most commonly observed deficiency alongside CI deficiency^8–10^.

In patients with mitochondrial myopathy, COX activity is heterogeneous between cells, with a mosaic pattern of enzyme deficiency where both COX normal and deficient fibres are present in the same biopsy, and this is easy to observe using histology. Mitochondrial dysfunction also exhibits a segmental pattern within muscle fibres, with dysfunctional mitochondria surrounded by healthy mitochondria, both longitudinally and in the transverse orientation^11,12^. Furthermore, mtDNA mutations can impair Ca^2+^ handling^13^, mitochondrial dynamics^14^ and patients with mtDNA mutations are found to have altered mitochondrial morphology and cristae organisation in skeletal muscle biopsies^2,15^.

Mitochondrial function and mitochondrial morphology are known to be intricately linked, with distinct morphologies associated with functional specialisation in some cell types and subcellular locations^16^. Mitochondrial morphology and dynamics play a role in regulating mitochondrial functions such as calcium homeostasis and apoptosiss^17^, are found to be associated with site of mitochondrial replication^18,19^ and are important for the mixing of mitochondrial contents and communication. Further, mitochondrial dysfunction leading to reduced oxidative phosphorylation causes a depolarisation of the mitochondrial membrane and mitochondrial fission^20,21^. In disease states specific mitochondrial structures are observed e.g. nanotunnels^15,22^ and donut mitochondria.

In a recent study by Vincent et al. ^2^, mitochondrial morphology and network organisation in human skeletal muscle were quantitatively analysed. Mitochondrial disease patients with mtDNA mutations were found to have an elevated frequency of simple mitochondria and mitochondrial nanotunnels^2^. However, the limitation of this work was that OXPHOS deficiency could not be detected, so any link between mitochondrial morphology and function could not be observed. Thus, it is unclear how mitochondrial morphology changes as OXPHOS deficiency progresses from normal through intermediate to deficient and finally to ragged red fibres (RRF), or what is happening in 3D space regarding the spread of dysfunction.

The successful development of the COX-SBFSEM technique on mouse tissue^23^ now allows us to apply this to patient tissue and correlate mitochondrial function and morphology in situ. This study aimed to extend the work previously done in SBF-SEM and use the COX-SBFSEM technique to compare OXPHOS-deficient muscle fibres with normal muscle fibres in mitochondrial disease patients.

## Results

### COX deficiency in patient muscle

**Fig. 1:**
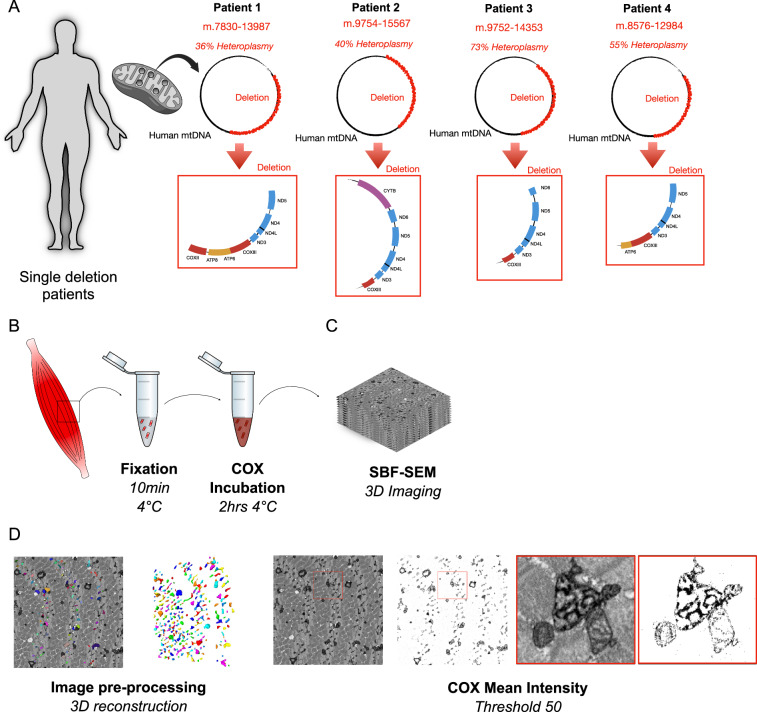
Schematic showing work flow for experiments. **A** Schematic of study design involving single deletion patients. **B** Muscle biopsy and teasing fibre. fixation and imaging of bundles muscle fibre and mitochondria in transversal orientation. **C** Z stack at EM resolution from serial block face scanning electron microscopy (SBF-SEM) used for 3D reconstructions (dimensions of 20 μm × 20 μm). **D** Image processing and 3D reconstruction of individual mitochondria (left) and thresholding for COX intensity including high resolution image of COX positive and negative mitochondria (left).

Patients 2, 3 & 4 would be classified as a class I deletion, based on work by Rocha et al. ^24^, because their deletions lie between CYB and ATP6 (Fig. 1A). Importantly, these patients will therefore have fibres that tend to have a combined complex I and IV deficiency. In comparison Patient 1 has a deletion that would be classified as a class III deletion, as this deletion extends into COXII (Fig. 1A), and we would expect fibres with isolated complex IV deficiency and fibres with both complex I and IV deficiency^24^, therefore the majority of OXPHOS deficient cells and mitochondria in all patients should be picked up as COX deficient by the COX-SBFSEM assay.

### COX activity at a single mitochondrion level

**Fig. 2:**
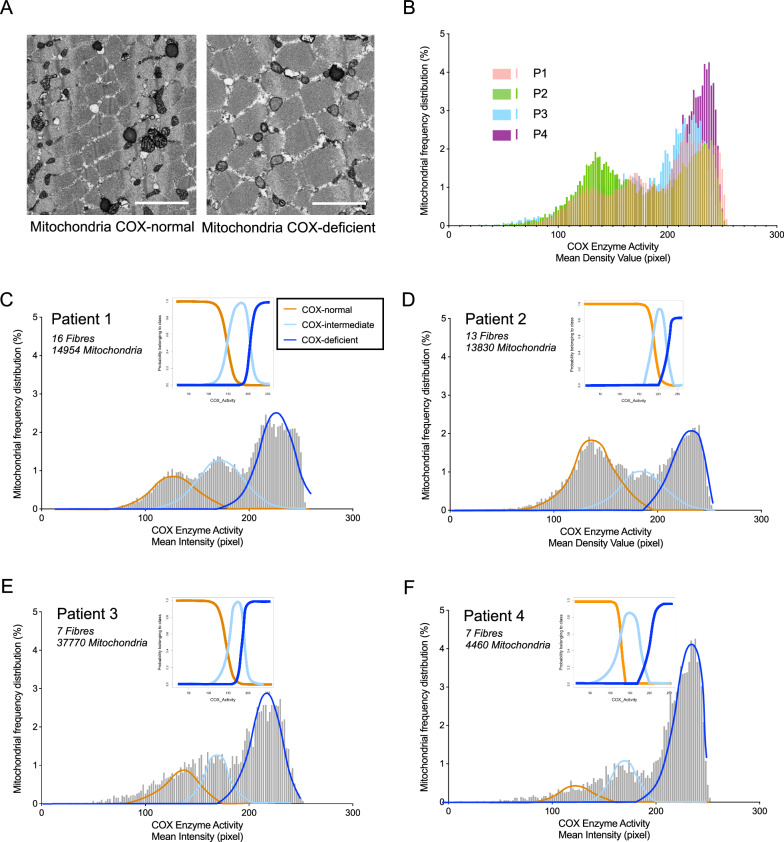
Mitochondrial COX activity falls into three distinct groups. **A** Single SBF-SEM image of a COX normal fibre (left) and a COX-deficient fibre (right). **B** Frequency distribution of mitochondrial COX activity from individual fibres of all single, large-scale mtDNA deletion patients. Patients, *n* = 4; Fibre, *n* = 43; Mitochondria, *n* = 37,014. Gaussian Mixture Model to P1 (**C**), P2 (**D**), P3 (**E**), P4 (**F**) data, each mitochondrion belongs to the orange, light blue, or blue cluster fit to COX activity distributions from Patient 1: Fibres *n* = 16; Mitochondria; *n* = 14,954. Patient 2: Fibres *n* = 13; Mitochondria; *n* = 13,830. Patient 3: Fibres *n* = 7; mitochondria *n* = 770. Patient 4: Fibres *n* = 7; mitochondria *n* = 4460.

The fibres to be imaged were selected by eye to yield a mix of COX-normal, -intermediate and -deficient fibres. COX normal fibres were easily recognisable by the dark, dense precipitate within the mitochondrial cristae, in comparison COX deficient fibres lacked this precipitate (Fig. 2A). The spectrum of COX intensity of individual mitochondria was analysed for each fibre (Fig. S1). An intensity value of 0 indicates black or electron dense, corresponding to a high concentration of COX and 255 indicates white or absence of the COX enzyme. For all patients, three groups of fibres grouped by their mean COX intensity are visible (Fig. 2B–F). The comparative COX intensity distributions for all patients are shown in Fig. 2B, highlighting the heterogeneity in distribution between patients.

Using Gaussian mixture modelling, an unsupervised clustering method, based solely on COX intensity, the mitochondria were split into three clusters (orange, light blue, or blue in Fig. 2C–F). A probability of each mitochondrion belonging to the orange, light blue, or blue cluster was calculated, the intersection between these curves represents the mitochondria where there is some uncertainty in classification and so a proportion of mitochondria cannot be classified (Fig. 2C–F). In order, to not lose large numbers of mitochondria, the intersection between the orange and light blue lines was used as a threshold between COX-normal and COX-intermediate mitochondria and the intersection between the light-blue and blue lines as the threshold between COX-intermediate and COX-deficient mitochondria, thus allowing us to classify all mitochondria as either COX-normal, -intermediate or -deficient.

### COX activity at the whole fibre level

**Fig. 3:**
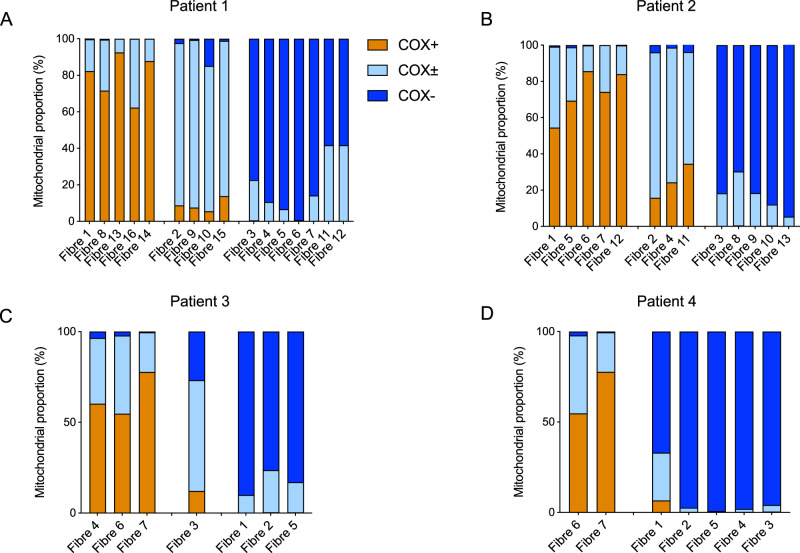
Proportion of mitochondria classified as COX positive, intermediate or deficient in each fibre. Proportion of Mitochondria classified as COX positive, intermediate and deficient within each fibre from **A** Patient 1, **B** Patient 2, **C** Patient 3 and **D** Patient 4. COX positive mitochondria are represented in orange; COX intermediate mitochondria are represented in light blue; COX negative mitochondria are represented in blue. Typically, fibres exhibiting more than 50% COX positive mitochondria were visually classified as COX positive fibres, those exhibiting more than 50% COX intermediate mitochondria were visually classified as intermediate fibres and those exhibiting more than 50% COX deficient mitochondria were visually classified as COX deficient mitochondria. Patient 1: Fibres *n* = 16; Mitochondria *n* = 14,954. Patient 2: Fibres *n* = 13; Mitochondria *n* = 13,830. Patient 3: Fibres *n* = 7; Mitochondria *n* = 3770. Patient 4: Fibres *n* = 7; Mitochondria *n* = 4460.

It is interesting to note that as expected, when we compared the proportion of COX normal, intermediate, and deficient mitochondria in each fibre there is a distinct pattern. In the fibres classified as COX positive or deficient by eye (Fig. 3), they generally have a greater proportion of COX+ or COX- mitochondria, respectively. Therefore, the fibre classifications were more quantitatively defined based on the percentage for each class of the individual mitochondria in a fibre (Fig. 3 and Table S1).

### The relationship between COX activity and mitochondrial morphology within muscle fibres

**Fig. 4:**
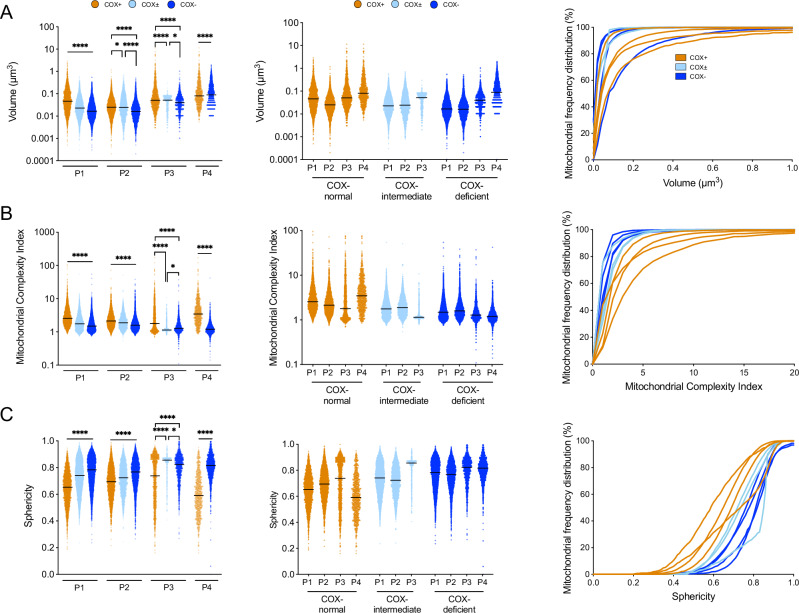
General morphological comparison between mitochondria of COX-normal, intermediate, and deficient fibres from all patients. Mitochondrial volume (**A**), MCI (**B**), and sphericity (**C**) with their respective cumulative frequency distribution of the COX-normal, COX-intermediate, and COX-deficient fibres. For each parameter 2 additional graph have been added: The first column displays median values for each metric, the second column shows patient-grouped data, and the third column presents the frequency distributions. Mitochondrial complexity Index (MCI) is a three-dimensional metric of mitochondrial shape complexity. MCI is an analogous to sphericity and scales with mitochondrial shape complexity, including branches and increased surface area relative to volume. Patient 1: Fibres *n* = 16; Mitochondria *n* = 14,954. Patient 2: Fibres *n* = 13; Mitochondria *n* = 13,830. Patient 3: Fibres *n* = 7; Mitochondria *n* = 3770. Patient 4: Fibres *n* = 7; Mitochondria *n* = 4460. Data are presented as median with 95% CI. Kruskal–Wallis test followed by post-hoc tests using the two-stage step-up method of Benjamini, Krieger, and Yekutieli to correct multiple comparisons (*p* < 0.05, *q* < 0.05).

Since previous work in cell models has found links between mitochondrial morphology and function, we sought to compare mitochondrial morphology between COX-normal, COX-intermediate and COX-deficient fibres across the four patients. The mitochondria from COX-normal fibres showed a large spectrum of volume and complexity in the four patients (*p < 0.05,****p < 0.0001, Fig. 4A–C). Concerning the COX-deficient fibres, mitochondrial volume was significantly smaller in Patients 1-3 but greater in Patient 4 (Fig. 4A, B).

Based on MCI, mitochondria were significantly simpler in COX-deficient fibres compared to COX-normal and -intermediate, with the exception of COX-intermediate fibres in Patient 3 which were slightly simpler than COX-deficient fibres (Fig. 4B). Interestingly, combined COX-deficient fibres for each patient showed average mitochondrial sphericity greater than 0.6 (Fig. 4C). Further, the proportion of spherical/morphologically simpler mitochondria that are COX-deficient (based on COX-deficient mitochondria for each patient representing 100%) are as follows: Patient 1: 67.8%, Patient 2: 50.4%, Patient 3: 46.6%, and Patient 4: 82.3%.

These percentages indicate a varying degree of morphological simplicity and sphericity among COX-deficient mitochondria across the different patients. The proportion of COX-deficient mitochondria that are morphologically simpler and spherical varies among patients, ranging from 46.6% to 82.3%. This suggests that while there is a general trend of COX-deficient mitochondria being more likely to be morphologically simple and spherical, the extent of this trend can differ between individuals and deletions. Simpler, more spherical mitochondria are associated with altered mitochondrial dynamics and could reflect an adaptive response to impaired OXPHOS function, potentially aiming to isolate the dysfunction from the mitochondrial network.

This finding supports our initial observation that COX-deficient mitochondria tend to be more morphologically simple and spherical compared to COX-normal mitochondria. This correlation suggests that mitochondrial sphericity could be a morphological marker for assessing the OXPHOS state and COX deficiency in mitochondria.

### Trends in COX activity and mitochondrial morphology across muscle fibres

**Fig. 5:**
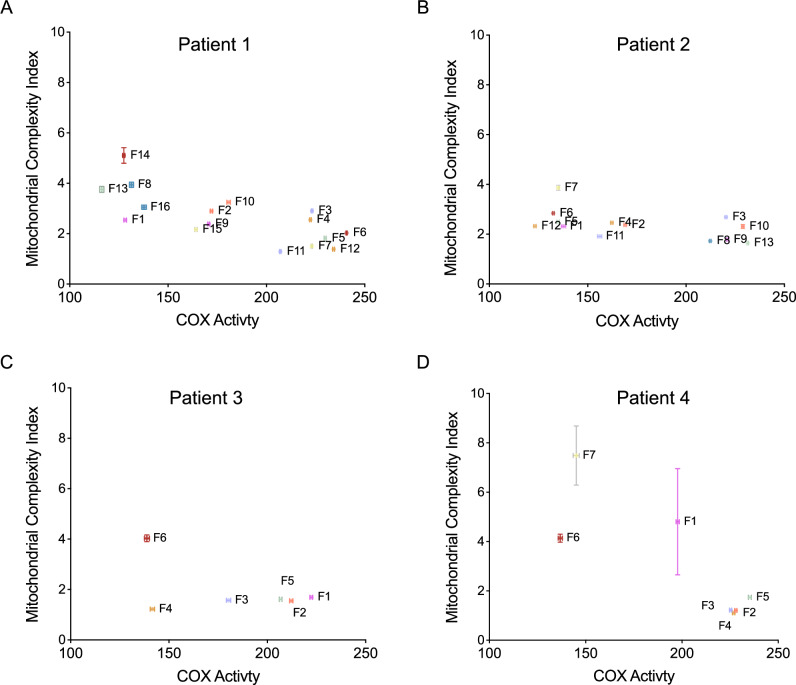
Effect of Single deletion mutation on mitochondrial morphology and function for each patient. Mitotypes illustrating the difference in COX activity and MCI between fibres (mean ± SEM), for patient 1 (**A**), patient 2 (**B**), patient 3 (**C**) and patient 4 (**D**). Patient 1: Fibres *n* = 16; Mitochondria *n* = 14,954. Patient 2: Fibres *n* = 13; ; Mitochondria *n* = 13,830. Patient 3: Fibres *n* = 7; Mitochondria *n* = 3770. Patient 4: Fibres *n* = 7; Mitochondria *n* = 4460.

We next wished to look at the relationship between the COX activity values for each fibre and the MCI values for each fibre. We did this by plotting the mean COX activity and SEM against the mean MCI and SEM for each fibre (Fig. 5). In Patient 1 it appears that MCI tends to increase as the COX activity increases (value decreases) (Fig. 5A). In Patient 2 this is also true however the range of MCI values is relatively narrow and so the relationship does not appear as strong (Fig. 5B). In Patient 3 we see the data distributed in an L shape with a cluster of fibres with similar MCI and a range of COX activity and then a few fibres with higher COX activity and a range of MCI values which are higher than for the other group of fibres (Fig. 5C). In patient 4 we can also see the pattern of MCI increasing with COX activity (Fig. 5D). These patterns suggest a strong relationship between mitochondrial complexity and COX activity in most patients. In Patient 3, fibre 4 appears to be a bit of an outlier. However, given that patient 3 has a deletion encompassing only one COX gene and five complex I genes, it may be that fibre 4 is complex I deficient but COX positive and therefore has a lower complexity due to respiratory chain deficiency not detected by the COX-EM assay.

### Multivariate analysis of mitochondrial morphology

**Fig. 6:**
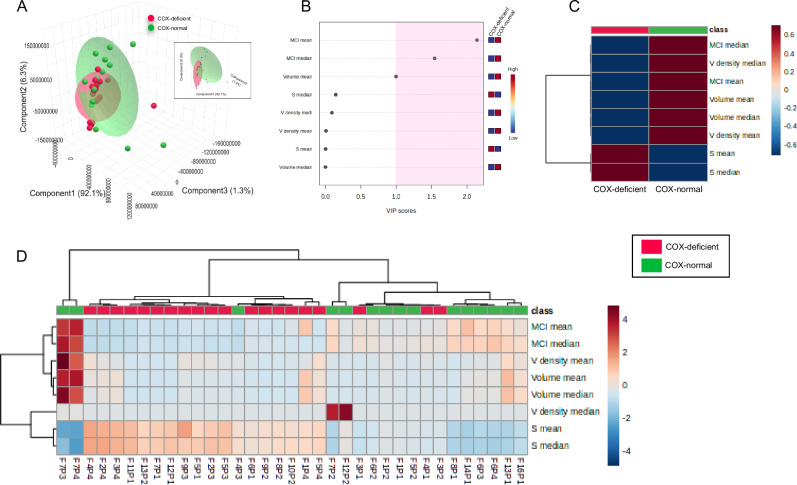
Multivariate analysis of mitochondrial morphology between individual normal and deficient fibres from all patients. **A** Partial Least Squares Discriminant Analysis (PLS-DA) highlights morphological variables specific to the differentiation of COX-normal and COX-deficient fibres. On the left, the PLS-DA score plots were presented for each type of variable. The model explains 92.1% (PC1) + 6.3% (PC2) + 1.3% (PC3) = 99.7% of the variance. This method is used to discern differences between different group. PC1 is the most significant component, capturing the majority of the variance (92.1%). This means that the majority of the morphological differences between COX-normal and COX-deficient fibres are explained by PC1. PC2 and PC3 contribute less to the variance but still provide additional information for differentiation. The COX-normal fibres are more widely distributed, with the COX-deficient fibres mostly contained within the red ellipse, with the exception of 3 points falling outside. Inset shows 3D PLS-DA from an alternative angle to more clearly see the separation of points. **B** The variable importance in projection or VIP score for the morphology parameters used in the PLS-DA regrouping the 4 patients together. The VIP score of a variable is a measure of its contribution to the model across all components. Higher VIP scores indicate greater importance in differentiating between classes. A common threshold for considering a variable important is a VIP score greater than 1. Variables with VIP scores above this threshold are typically considered significant in the model. The coloured boxes on the right indicate the relative concentrations of the corresponding metabolite in each group under the current study. Heatmap across group average (**D**), and all subjects and parameters (**C**), with dendrograms illustrating hierarchical clustering of pattern similarity across morphological parameters and samples (top) (Euclidean distance measure, Ward clustering algorithm). The colours indicate the relative quantitative value, where red indicates a higher value, and blue indicates a lower value. The COX-normal fibres: Patient 1: *n* = 5; Patient 2: *n* = 5; Patient 3: *n* = 3; Patient 4: *n* = 2. The COX-deficient fibres: Patient 1: *n* = 7; Patient 2: *n* = 5; Patient 3: *n* = 3; Patient 4: *n* = 5.

Previously Vincent et al. ^2^ performed multivariate analyses and demonstrated that mitochondrial morphology could be used to distinguish mitochondrial disease patients and controls. As such, we sought to determine whether similar approaches could be used to distinguish COX-positive, -intermediate and deficient fibres. We applied multivariate analysis methods similar to those used by Vincent et al. ^2^. Using Partial Least Squares-Discriminate Analysis, which is a method of multidimensionality reduction used to discern differences between different groups or classes of data points. PLS-DA in three dimensions, the first of which captured 99.7% of the total variance in the dataset (Fig. 6A). The COX-normal fibres were more widely distributed, with the COX-deficient fibres mostly contained within the red ellipse, with the exception of 3 points falling outside (Fig. 6A). For each parameter, the rank ordering of the variable importance in projection (VIP) scores (a score >1 is considered significant) identified the most important morphological features that differentiate between COX-normal and COX-deficient fibres (Fig. 6B). The top four features for all patients together when comparing COX positive vs negative fibres, were (1) the MCI mean, (2) the MCI median, (3) the volume mean and (4) the sphericity median (Fig. 6B), where the first 3 had highest values for COX normal fibres. The top features were also generated for each patient separately, similarly, to compare COX normal vs deficient fibres (Fig. S2), demonstrating some clear similarities and differences between patients. MCI mean was important for all but Patient 4, whereas MCI median was important for all but Patient 1. For all patients combined a heat map was generated based on group means of each fibre classification, COX-deficient fibres had a higher sphericity compared to the COX-normal and other parameters were all lower for all patients combined (Fig. 6C) and for each patient individually. This suggests that COX-deficient mitochondria are more spherical, simpler and smaller than COX-positive mitochondria (Fig. 6C).

Hierarchical clustering highlights the similarities and differences between COX-normal and COX-deficient fibres (Fig. 6D). This unsupervised analysis which clusters fibres based on similarities between samples and functional parameters shows that COX-normal vs COX-deficient fibres mostly segregate into two distinct clusters. One exception came from three COX-deficient fibres, which had lower mitochondrial sphericity (Fibre 3 Patient 1, Fibre 4 Patient 1, Fibre 3 Patient 2) than the other deficient fibres (Fig. 6D) and three COX-normal fibres two of which have much higher volume, MCI and volume density than any other fibres and one of which has a higher sphericity than the other COX-normal fibres. The two fibres with much higher volume, MCI and volume density appear to have been classified differently from both groups and may present RRF.

### The relationship between COX activity and mitochondrial morphology in individual mitochondria

**Fig. 7:**
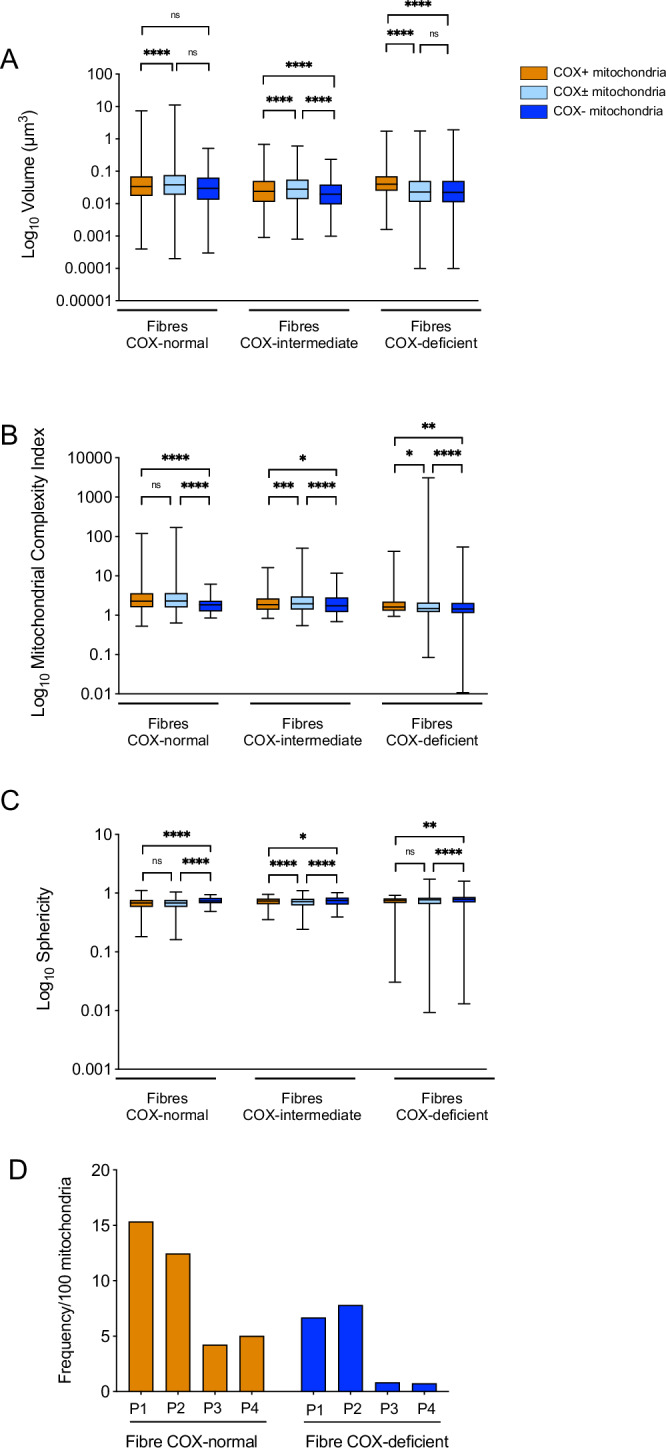
Morphological comparison of the three distinct mitochondria groups present within each fibre. This involve analysis of the COX-normal, COX-intermediate, and COX-deficient fibres of all the regrouped patients in order to draw a conclusive comparison. **A** Box and whisker plots for mitochondrial volume, MCI (**B**), and sphericity (**C**) of the COX-normal, COX-intermediate, and COX-deficient fibres. Box and whisker represent median, 25th quartile and 75th quartile as well as minimum and maximum values. **D** Nanotunnels frequency (/100mitochondria) quantification for one fibre COX-normal and one fibre COX-deficient per patient. Patient 1: Fibres *n* = 16; Mitochondria *n* = 14,954. Patient 2: Fibres *n* = 13; Mitochondria *n* = 13,830. Patient 3: Fibres *n* = 7; Mitochondria *n* = 3770. Patient 4: Fibres *n* = 7; Mitochondria *n* = 4460. Data are presented as median with 95% CI. Kruskal–Wallis test followed by post-hoc tests using the two-stage step-up method of Benjamini, Krieger, and Yekutieli to correct multiple comparisons (*p* < 0.05, *q* < 0.05).*p < 0.05,**p < 0.005, ***p < 0.0005, ****p < 0.0001.

We initially compared the morphology of COX-normal, intermediate, and deficient mitochondria within normal, intermediate and deficient fibres (Fig. 7A–C). Within normal fibres from all patients, the COX-normal mitochondria were on average significantly larger and more complex, than COX-negative mitochondria, which were more spherical (Fig. 7A–C). Regarding the intermediate fibres, the COX-intermediate mitochondria were larger and more complex, while the COX-deficient mitochondria were smaller and more spherical within these fibres. In addition, within the deficient fibres, COX-deficient mitochondria were also smaller and more spherical than the others. However, when looking at individual patients, no significant differences were seen in MCI between these 3 groups for individual patient, however, the N was too small to draw a robust conclusion (Figs. S3–S6).

### COX deficiency and mitochondrial morphology with spatial resolution

**Fig. 8:**
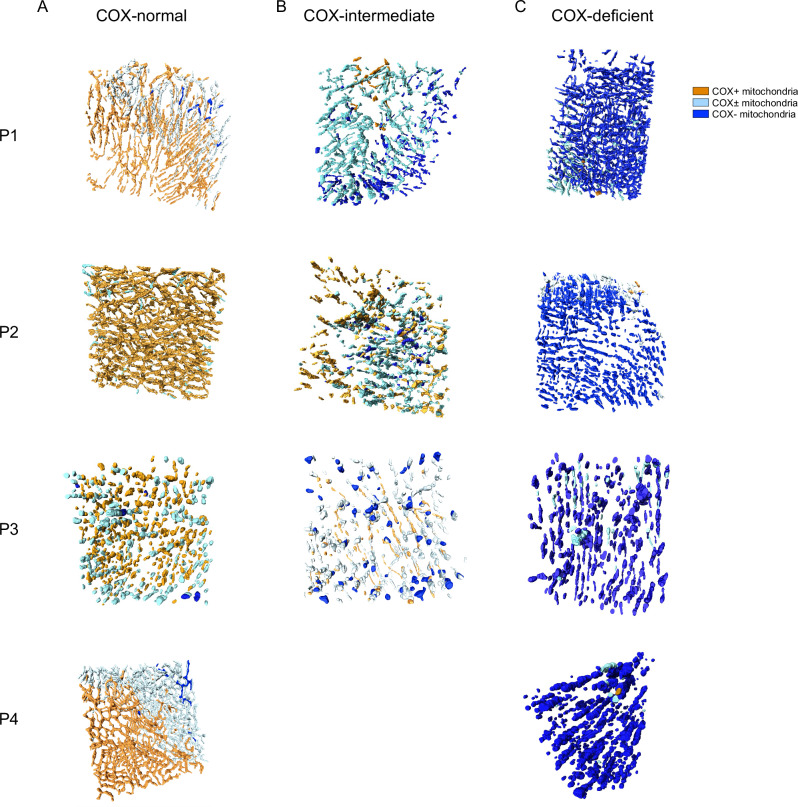
COX activity and spatial distribution of the mitochondrial COX activity and their respective morphologies within a COX-normal/COX-intermediate and COX-deficient fibres from Patient 1,2,3&4. The 3D reconstruction figures of a COX-normal (Fig. 7A), COX-intermediate (Fig. 7B) and COX-deficient (Fig. 7C) fibre demonstrate coloured mitochondria according to their COX activity. 3D reconstruction of mitochondrial COX activity across two sarcomeres in COX normal (**A**), COX-intermediate (**B**) and COX-deficient (**C**) fibres demonstrate coloured mitochondria according to their COX activity for Patients 1-4. Mitochondria are colour coded dependent on COX activity as indicated by the scale Cluster of COX-normal and intermediate mitochondria can be distinguished by the dotted circle.

The intracellular COX heterogeneity distinguished above is illustrated in Fig. 8. Within the COX-normal fibre, COX-normal and intermediate mitochondria are generally grouped spatially in a strong pattern (Figs. 8, and S7). This pattern can be also seen in the COX-intermediate fibre. Interestingly, most of the COX-deficient fibres exhibited a mix of COX-deficient and intermediate mitochondria, with and without a spatial pattern.

**Fig. 9:**
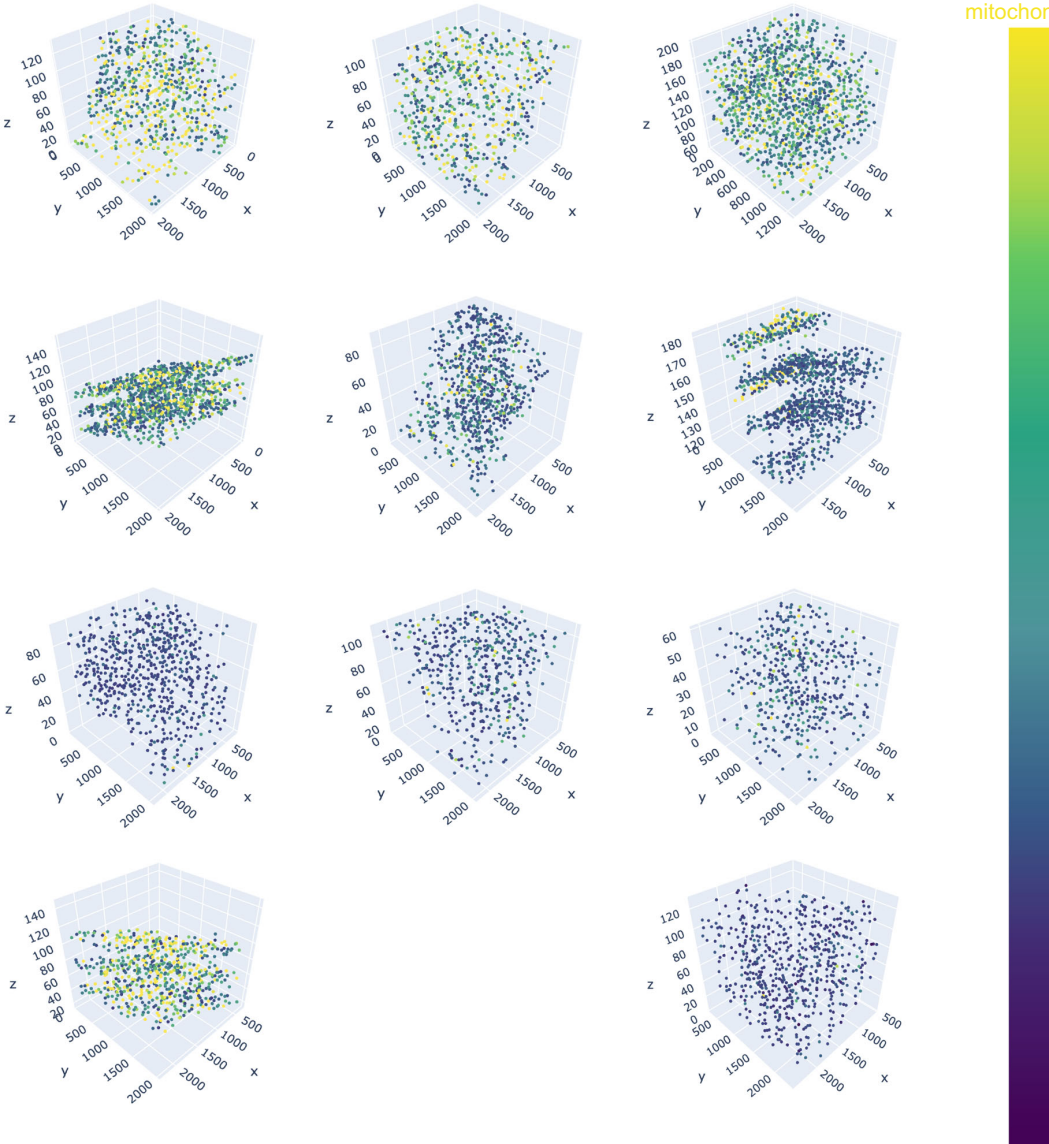
Mitochondrial spatial distribution and their MCI within a COX-normal/COX-intermediate and COX-deficient fibres from all patients. 3D scatter plot of mitochondrial complexity index across two sarcomeres in COX normal (A), COX-intermediate and COX-deficient fibres. Mitochondria are colour coded dependent on mitochondrial complexity index as indicated by the scale.

When we look at mitochondrial morphology using MCI in a similar way, we find very little pattern spatially in COX-normal, COX-intermediate and COX-deficient fibres (Fig. 9). However, to better visualise the morphology and COX activity together we generated videos of the fibres in Figs. 8 and 9 where the 3D reconstruction has been colour-coded based on COX activity (COX-normal: orange, COX-intermediate: light blue, COX-deficient: dark blue). In Video 1 you can see a COX normal fibre from Patient 1 which has highly complex normal mitochondria with a small number of more ed deficient mitochondria, with a strong spatial pattern to mitochondrial function. In Video 2 we present the intermediate fibre from Patient 4, which shows a strong spatial pattern regarding their activities and a mix of mitochondrial morphologies, where the COX deficient mitochondria still exhibit simple mitochondrial morphology. Finally, in Video 3 we present a COX deficient fibre from Patient 2, where most mitochondria are deficient and relatively simple in morphology but a strong spatial pattern in COX activity is visible.

### Interaction with lipid droplets

Previous work has noted the interaction of mitochondria and lipid droplets in skeletal muscle^25,26^. However, due to the lack of methods it has not been possible to observe whether there is a change in the interactions with mitochondrial dysfunction. Here, using COX-SBFSEM, we observe that mitochondria-lipid droplets interactions are most observed in muscle fibres that are COX-deficient.

## Discussion

This study investigated the spatial distribution of COX activity and morphology of mitochondria in muscle biopsies of patients with mitochondrial myopathy using volume-EM. Previous work in cultured cells has demonstrated that a relationship exists between mitochondrial morphology and function^27–30^. In addition, in muscle biopsies from patients with mtDNA disease, mitochondria appeared damaged, more fragmented^2^ and also exhibited aberrant cristae^15^. However, it was necessary to have an assay capable of detecting both function and morphology, to be able to assess the relationship between these in patient tissue.

Using our COX-SBFSEM assay we present the analysis of both mitochondrial morphology and function in human muscle biopsies. These biopsies are from patients with single, large-scale mtDNA deletions, which will be present in individual muscle fibres at different levels of heteroplasmy, leading to a mosaic pattern of mitochondrial OXPHOS deficient and normal muscle fibres. Across all mitochondria presented here we generally find that lower COX intensities or activities are associated with smaller mitochondrial complexity index (MCI) values and vice versa. Similar findings have been reported in our previous work where higher levels of m.8344A>G mutation in patient muscle are associated with more fragmented mitochondria, however due to the lack of COX activity measurements no direct correlation between OXPHOS and morphology was possible in that study^2^. This also fits with previous reports from cybrids where a high level of mtDNA heteroplasmy which will be associated with mitochondrial dysfunction, leads to fragmentation of the mitochondrial network^31^. However, interestingly in the cybrids a medium level of mutation load which would be associated with lower levels of mitochondrial dysfunction are associated with more branched mitochondria. This is at odds with what we see here, however since all previous findings that have been able to observe mitochondrial function and morphology simultaneously have not used patient muscle samples, it is likely this may present an important difference between human muscle and previously studied models.

Another interesting observation from our ability to look in situ is that each fibre is in fact a mix of mitochondria with different COX activities. Confocal and electron microscopy have revealed heterogeneous COX activity and mitochondrial membrane potential, with an altered morphology for the COX deficient mitochondria in individual cells derived from clonal heteroplasmic cybrid cell lines with the m.3243A>G mutation^32^. This fits with the mixture of COX activities we observe here in a single cell since the mitochondrial membrane potential related to the function of the respiratory chain and also impacts to mitochondrial morphology^33,34^. Vincent et al. ^2^ also showed that mitochondrial morphological heterogeneity is demonstrated by the coexistence of a wide variation in morphology within a cell or patient^2^. The presence of a moderate level of mtDNA heteroplasmy in mitochondrial disease was associated with more heterogeneity^2^. Here, the results indicate a heterogeneous population of complex and simple mitochondria inter- or intracellularly in patient muscles for all patients. Perhaps most intestinally in a group of patients that are renowned for their heterogeneity similar patterns are observed across all four patients, including the simpler spherical COX-deficient mitochondria when compared to the COX normal mitochondria and the proportion of mitochondria with different COX activities in the different classes of fibres.

Within the COX-deficient fibres, mitochondria also appeared to be simpler with a lower MCI in all patients, whereas COX-normal fibres exhibit a higher MCI. The mitochondrial fragmentation exhibited in COX-deficient fibres, suggest that the dysfunction is too high and as such is undergoing a stress-induced mitochondrial fragmentation, similar to previous findings^2^. This fragmentation may occur due to changes in membrane potential, ATP levels or as a precursor to mitophagy, although mitophagy has recently been shown to be stalled in a mitochondrial myopathy^35^. Alternatively, in fibres with low levels of dysfunction, such as COX-normal and intermediate fibres, mitochondrial fusion may restore homeostasis, promote mtDNA mixing and act as a buffer for mitochondrial dysfunction, which is also supported by previous publications finding increased fusion at low levels of heteroplasmy or metabolic stress^30,36,37^.

Within a segment of skeletal muscle fibre, a proportion of mutated mtDNA may exceed a critical threshold, resulting in COX deficiency^38^. When mtDNA variants reach a high mutation load due to intracellular clonal expansion, a biochemical defect results in COX-deficient mitochondria, which can be restricted to spatially resolved segments of the muscle fibre in both the longitudinal and transverse orientations, contributing to the appearance of clinical pathology and disease progression^39^. Chinnery, et al. ^40^ provide objective evidence that the clinical progression of mtDNA myopathy is associated with a biochemical deficiency that develops independently within individual muscle fibres. To understand how the mtDNA variant proliferate and the spread of the deficiency during clonal expansion, Vincent et al. ^12^ studied muscle fibres from patients with mitochondrial disease. The results from this study showed deficiency to occur in focal regions surrounding the myonuclei, inducing mitonuclear signalling and leading to proliferation of the dysfunctional mitochondria locally before spreading through the muscle fibre. Further work demonstrated that the deficient mitochondria will propagate transversely first via direct physical interactions between mitochondria before propagating longitudinally where fewer mitochondrial connections are observed^2,12^.

The EM data obtained here matches with previous reports of segmental COX deficiency within muscle fibres^12,41,42^. What is particularly interesting is that here we show that in normal fibres small numbers of isolated COX-deficient mitochondria are observed, this is somewhat consistent with previous work which was the first to describe a focal perinuclear niche of COX deficiency in otherwise normal fibres^12^. These structures whilst in frequent appear to present a common pathological mechanism for the origins and development of mitochondrial dysfunction in skeletal muscle across ageing, inclusion body myositis and mitochondrial myopathy.

**Fig. 10:**
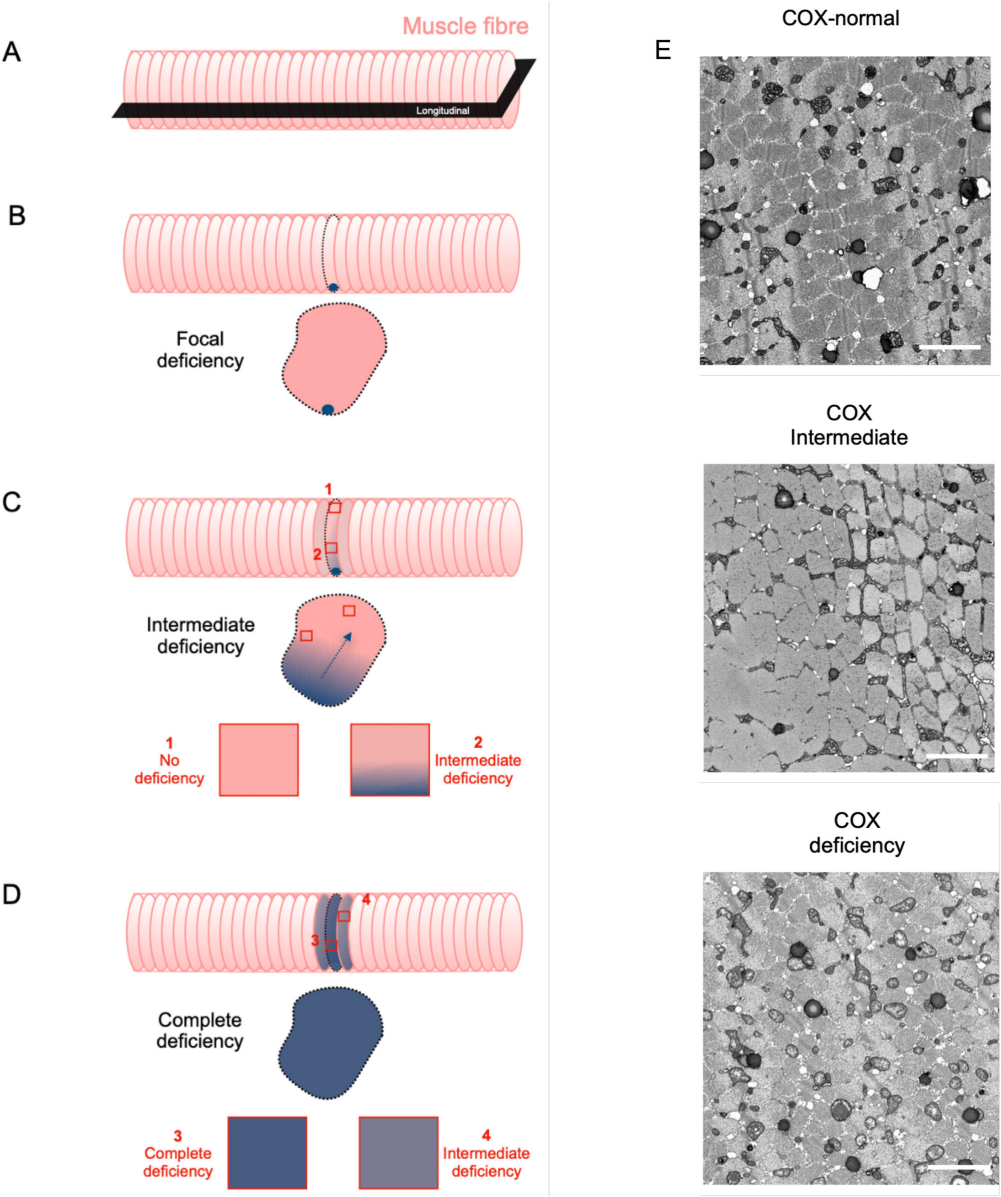
Model illustrating spread of COX-deficiency. **A**–**E** A theoretical guide to understanding and classifying the development of COX deficiency. Focal perinuclear deficiency (**B**) to segmental deficiency (**C**). The spread of the dysfunction involves COX-intermediate stages before the full complete COX-deficiency muscle fibre (**D**). Examples of 2D images from Fibres COX-normal, intermediate and deficient (**E**).

In intermediate fibres, we see a mix of mitochondrial activities and morphologies again with an obvious spatial pattern. Furthermore, intermediate mitochondria generally have a complexity that is somewhere between that of COX-normal and -deficient mitochondria and present the spatial interface between the two. This suggests to us that it is this intermediate stage where the spread of dysfunction is most likely to occur (Fig. 10). This would fit with previous findings, which demonstrate that at low levels of mtDNA heteroplasmy^2,31^ and metabolic stress^30,36^, mitochondria are complex and connected and therefore there is a greater opportunity to share material and for dysfunction to spread. Similarly, in COX normal muscle fibres that we have examined there appear to be small numbers of negative mitochondria with a strong spatial segregation of COX activity and a small region of intermediate mitochondria that act as a transition zone between the normal and deficient mitochondria (Fig. 10), again the COX-intermediate mitochondria likely representing a transition zone between the two. These fibres we believe to be important to informing our knowledge of how dysfunction spreads, with the intermediate mitochondria being the key players. However, when we look at the COX-deficient fibres, we see that the COX-deficient mitochondria have become isolated, but perhaps too little too late as the dysfunction has already spread to the neighbouring mitochondria.

We would be remiss not to consider an alternative hypothesis that these spatially restricted COX-deficient mitochondria, once dispersed, have segregated from the network and spatially over time. However, this would appear counter-intuitive with the vast literature demonstrating the clonal expansion of mtDNA deletions throughout life, associated with the worsening of OXPHOS dysfunction, segmental deficiency and proportion of fibres exhibiting OXPHOS dysfunction^42–44^. Such accumulation and spread of mitochondrial mtDNA mutations and dysfunction is observed without the spatial affects in other tissues^45–47^. However, unlike muscle fibres which are dense in cytoskeletal structure the same spatial pattern is not observed in other tissues, likely due to the rapid movement of mitochondria throughout the cell.

The findings of Murphy, et al. ^44^, using COX histochemistry, have demonstrated that intermediate levels of COX activity exist between COX-normal regions and fully COX-deficient segments of muscle fibres, which they term as COX-intermediate segments. This COX-intermediate segment effectively being the transition zone, between normal and deficient regions, where dysfunction is spreading along the fibre. Thus, this diverse range of mitochondrial COX activity could underlie how the COX deficiency within individual muscle fibres ranges from focal to segmental and complete deficiency in patients with mtDNA disease^12,48^(Fig. 10).

In this work we have aimed to understand the link between mitochondrial function and mitochondrial morphology in situ, however, it is worth noting an important limitation. Patients with mtDNA deletions will have deletions that remove different numbers of genes encoding subunits for complexes I, III, IV and V as well as tRNAs. As such it is possible that whilst our assay shows a muscle fibre or even a mitochondrion as being COX positive it may have a deficiency in one of the other complexes which we are unable to detect with this assay. However, the fact that we see clear differences between the different muscle fibre classes suggests to us this is a have only a small impact on the fibres analysed here.

A limitation here is that each ROI was imaged from the centre of a fibre. Therefore, a strong spatial pattern cannot easily be observed, as it is just a part of each individual fibre that is imaged and only a small depth through the fibre. However, these results may suggest that each fibre is at a particular stage of COX deficiency, due to the segmental spread of the dysfunction. Furthermore, for some fibres, the clusters were well-defined, especially with COX-normal, and intermediate fibres along the muscle fibre diameter. This is important in terms of progression and assessing mitochondrial disease severity. Alongside this, it is challenging to determine the type of muscle fibre type using electron microscopy, as the quadriceps muscle contains a mixture of muscle fibres with different properties.

This can be problematic because muscle fibres exhibit different mitochondrial volume density and morphology. In the future, detecting fibre types would allow for a better understanding, of the fibre type-specific differences. Further work to explore the relationship between COX activity and cristae morphology. We also acknowledge that a more extensive quantification of the frequency of nanotunnels would yield further insight into how the mitochondria adapt to reduced COX activity. However, the number of investigated fibres from each patient is a compromise between exploring enough to account for variability and feasibility due to the manual work required for accurate mitochondrial segmentation.

## Conclusions

This work provided the first quantitative assessment in single, large-scale mtDNA deletion patients’ skeletal muscle of mitochondrial morphology and COX function using SBFSEM. This study shows first the spectrum of COX activity within muscle fibres, with three distinct mitochondrial populations: normal, intermediate, and deficient. Secondly, it shows the distribution of mitochondrial dysfunction and associated changes in mitochondrial morphology, with COX deficiency associated with simple mitochondria. It is also interesting to observe some degree of spatial pattern, which seems to highlight a key role of intermediate mitochondria in the spread of mitochondrial dysfunction. Finally, this study gives insights into how mitochondrial function is linked to mitochondrial morphology in situ, which prior to this were technically impossible to measure.

## Methods

### Biopsy processing and Cohort clinical characteristics

Quadriceps muscle samples were collected through the AIMM Trial (AIMM Trial Group, *Trials* (2022); REC ref: 18/NI/0199, ISRCTN: 12895613). Samples for EM were dissected after biopsy and placed into fixative within 5 min. Baseline samples (~1 mg) from four patients with single, large-scale mtDNA deletions (Table 1) were processed for EM. Mutation load was determined as part of the AIMM Trial. Deletion breakpoints were confirmed from muscle DNA requested from the Newcastle Mitochondrial Research Biobank (NMRB050; REC ref: 21/NE/0204). Figure 1 shows schematically which genes are deleted in each patient.

**Table 1:** Clinical and genetic information for subjects included in this study

Patients	Sex	Age at biopsy	Genetic defect	Mutation load
**P1**	F	47 y	Deletion size ~ 6.1 kb Breakpoints: 7830–13987	36%
**P2**	F	43 y	Deletion size ~ 5.8 kb Breakpoints: 9754–15567	40%
**P3**	M	26 y	Deletion size: ~4.6 kb Breakpoints: 9752–14353	73%
**P4**	F	49 y	Deletion size ~ 4.5 kb Breakpoints: 8576–12984	55%

*f* female, *m* male, *y* years, *Kb* Kilobases.

### COX - SBFSEM processing and imaging

Human muscle samples were processed with the COX-SBFSEM method, as previously described^23^. Briefly, within 5 min after each muscle biopsy, muscle sample were put in a short fixation for 10 min at 4 °C, followed by incubation with COX medium for 2 h at 37 °C in the dark. Samples were fixed for 10 min at 4 °C and washed three times with Sorenson’s buffer in the microwave (3 × 50W 40 s per step as described by ref. ^23^). The samples were incubated with the COX reaction for 2 h at 37 °C in the dark^23^. The tissue was processed using a heavy metal protocol. Briefly, samples were subsequently fixed, and membranes were stained with 3% potassium ferrocyanide and 2% osmium tetroxide for 1 h at room temperature (RT). Following osmium fixation, the remainder of the phosphate buffer was removed by washing in 0.1 M Sorenson’s buffer (3 × 40 s per step, 150 W^23^). The samples were placed into a contrast enhancer 0.1% thiocarbohydrazide (TCH) filtered at RT for 20 min and immersed in 2% osmium tetroxide for 30 min at RT. Finally, samples were placed into 1% uranyl acetate overnight at 4 °C. On day two, all samples were washed in several changes of distilled water (ddH_2_O; 5 × 3 min RT and microwave 3 × 40 s 150 W^23^). Following these washes, immersion for 30 min at 60 °C in lead aspartate solution (previously heated for 30 min: 0.12 g of lead nitrate in 20 mL aspartic acid)^49^ was carried out. Dehydration was performed in a graded series of acetone from 25% to 100% and impregnation in increasing concentrations of Taab 812 hard resin (25% to 100%) in acetone with several changes to 100% resin. Samples were embedded in 100% fresh resin and left to polymerise at 60 °C for a minimum of 36 h (Fig. 1B). After polymerisation, the resin blocks were trimmed to ~0.75 mm × 0.75 mm and sectioned as described by ref. ^23^ to identify the regions of interest (ROIs) before performing SBFSEM^23^.

For each sample, transversely orientated muscle fibres were visually selected to have as many COX-normal and COX-deficient fibres in close proximity to each other as possible (i.e. minimise re-localisation and imaging time). One ROI for IMF mitochondria was selected for each fibre and imaged. The samples were sectioned and captured in a series of images (400 images per stack) at 70 nm sectioning thickness. The image resolution achieved with SBF-SEM was 2000 × 2000 pixels and a pixel size of 0.07 μm × 0.07 μm in the *x*,*y* dimensions^23^.

### Image analysis pre-processing and mitochondrial 3D reconstruction

Image stacks from Digital Micrograph (Gatan) were normalised as described in Faitg et al. ^23^ and converted to Tiff files. Briefly a standard operation, which consists of 3 steps: (i) obtain the mean and standard value of intensities for the whole dataset^50^, for each image, and finally (iii) shift/stretch each image such that its mean/standard values matched the mean/standard of the whole dataset^23^. Once in the right format, the mitochondrial segmentation was completed throughout the stack in all three dimensions, across two sarcomeres from A-band to A-band, to generate 3D reconstructions (MIB, Helsinki version 2.702^51^). The mitochondria were manually traced using the ‘brush’ tool in MIB software^51^. After which, the mitochondria were reconstructed in 3D to extract volume and surface area data with Amira version 2020.3^52^. Mitochondrial Complexity Index (MCI): [MCI = ((SA^1.5^)/4πV)^2^] a 3D equivalent to form factor which quantifies mitochondrial shape complexity, was calculated as described previously^2^.

### Analysis of COX labelling intensity

The electron microscopy 3D images stacks (8-bit) were normalised altogether using MIB as mentioned above. For measurements of COX-specific activity, the threshold was manually set to include only COX-specific enzyme cytochemical product. An intensity threshold of 50 was chosen so that only the COX-specific precipitate and therefore only COX-positive cristae were visible (Fig. 1D). The mean intensity per pixel was obtained for each individual mitochondrion from all fibres. The mean intensity value was close to 0 for black and 255 for white.

### Proportion of spherical/morphologically simpler mitochondria that are COX-deficient

To better understand the relationship between COX activity and mitochondrial morphology we sought to look at the number of spherical mitochondria that are COX-deficient. We defined spherical mitochondria as having a sphericity of 0.75. We then calculated the percentage of COX-deficient mitochondria.

### Statistical analysis of mitochondrial morphology and COX-EM data

A normality test was performed on all datasets for the four patients and histograms plotted in Prism v9.0 (Graph Pad) to assess normality. The data were not normally distributed, precluding the use of parametric statistical tests. Therefore, for multiple comparisons (more than two groups) the Kruskal–Wallis test or Mann–Whitney (for two groups only) were applied, to examine the main effects, followed by posthoc tests using the two-stage step up method of Benjamini, Krieger, and Yekutieli to correct for multiple comparisons (*p < 0.05, *q* < 0.05).

### Multivariate analysis and machine learning

To describe the observed distributions of mitochondrial morphology from the four patients, partial least-squares discriminant analysis (PLS-DA) and clustering analysis using a hierarchical ward algorithm presented as a heatmap were generated using the MetaboAnalyst 5.0^53^. Partial Least Squares Discriminant Analysis (PLS-DA) highlights morphological variables specific to the differentiation of COX-normal and COX-deficient fibres

The variable importance in projection or VIP score for the morphology parameters used in the PLS-DA regrouping the 4 patients together. The VIP score of a variable is a measure of its contribution to the model across all components. The VIP scores were extracted for each mitochondrial feature for the PLS-DA models.

Higher VIP scores indicate greater importance in differentiating between classes. A common threshold for considering a variable important is a VIP score greater than 1. Variables with VIP scores above this threshold are considered significant.

### Neighbourhood analysis

To visualise whether deficient mitochondria cluster together, the x,y,z coordinates of every single mitochondrion and their respective COX activity were put together in the same 3D scatter plot. The source code can be found on github: https://github.com/AEVincent/Faitg-et-al or on Zenado^54^.

### Reporting summary

Further information on research design is available in the Nature Portfolio Reporting Summary linked to this article.

## Supplementary information

Supplementary Information

Description of Additional Supplementary Files

Supplementary data 1

Reporting summary

## Data Availability

All data, images and models are available on request. Numerical source data for all graphs in the manuscript can be found in Supplementary data 1 file.

## Code availability

Code available on github: https://github.com/AEVincent/Faitg-et-al or on Zenado^54^.
