# Supplementary material for: Mapping mitochondrial morphology and function: COX-SBFSEM reveals patterns in mitochondrial disease
Source: Commun Biol. 2025 Jan 9;8:24. doi: 10.1038/s42003-024-07389-7 (PMC11718190; doi:10.1038/s42003-024-07389-7)
Supplement: Supplementary file 1 — Supplementary Information [file 42003_2024_7389_MOESM1_ESM.pdf]

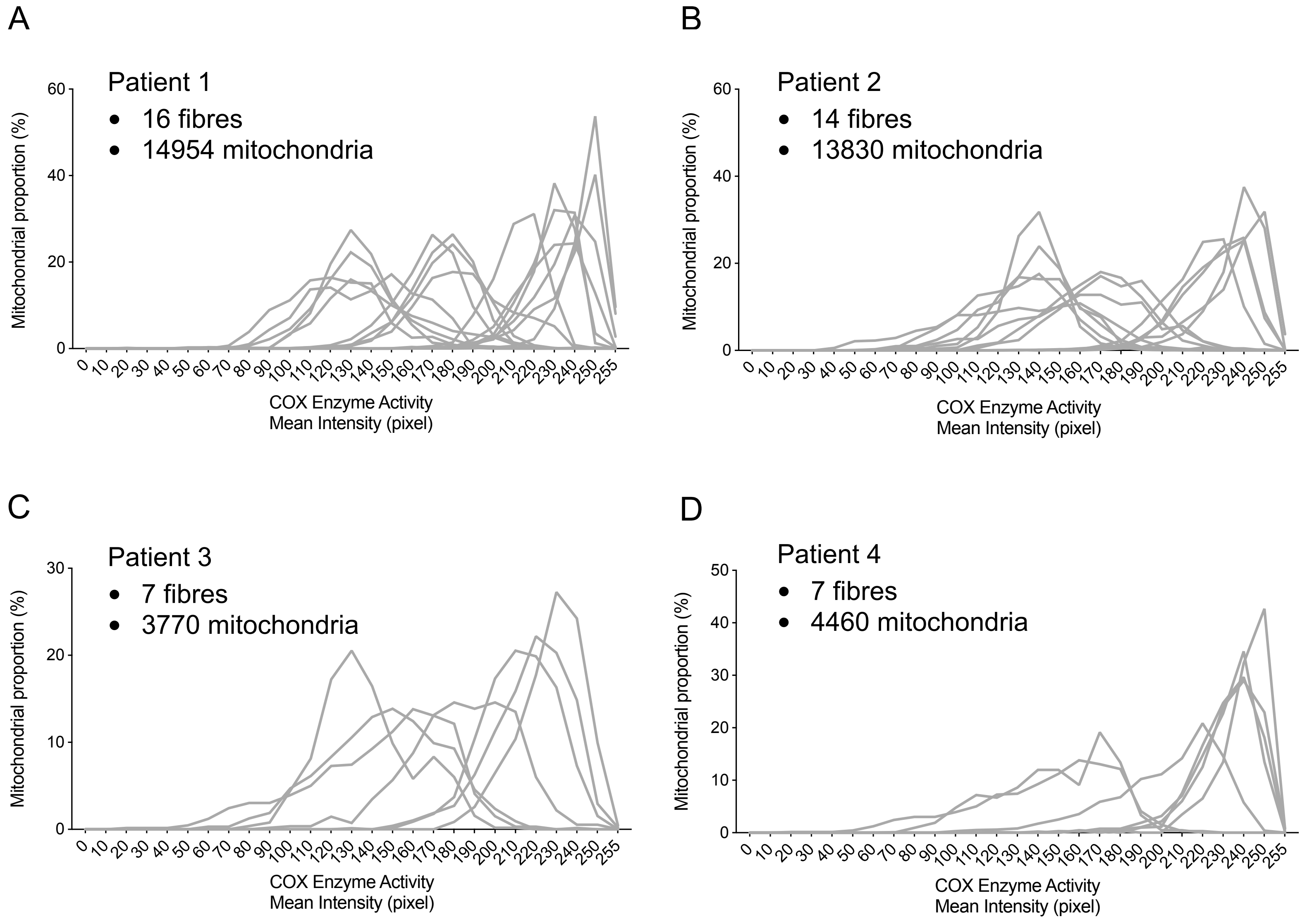

Figure S1. Mitochondrial COX activity for individual fibres.

Frequency distribution of mitochondrial COX activity from individual fibres of single, large-scale mtDNA deletion Patient 1 **(A),** Patient 2 **(B),** Patient 3 **(C)** and Patient 4 **(D).** The selection of the fibres was assessed by eyes.

Patient 1: Fibres n=16; Mitochondria n=14954. Patient 2: Fibres n=13; Mitochondria n=13830. Patient 3: Fibres n= 7; Mitochondria n=3770. Patient 4: Fibres n=7; Mitochondria n=4460.

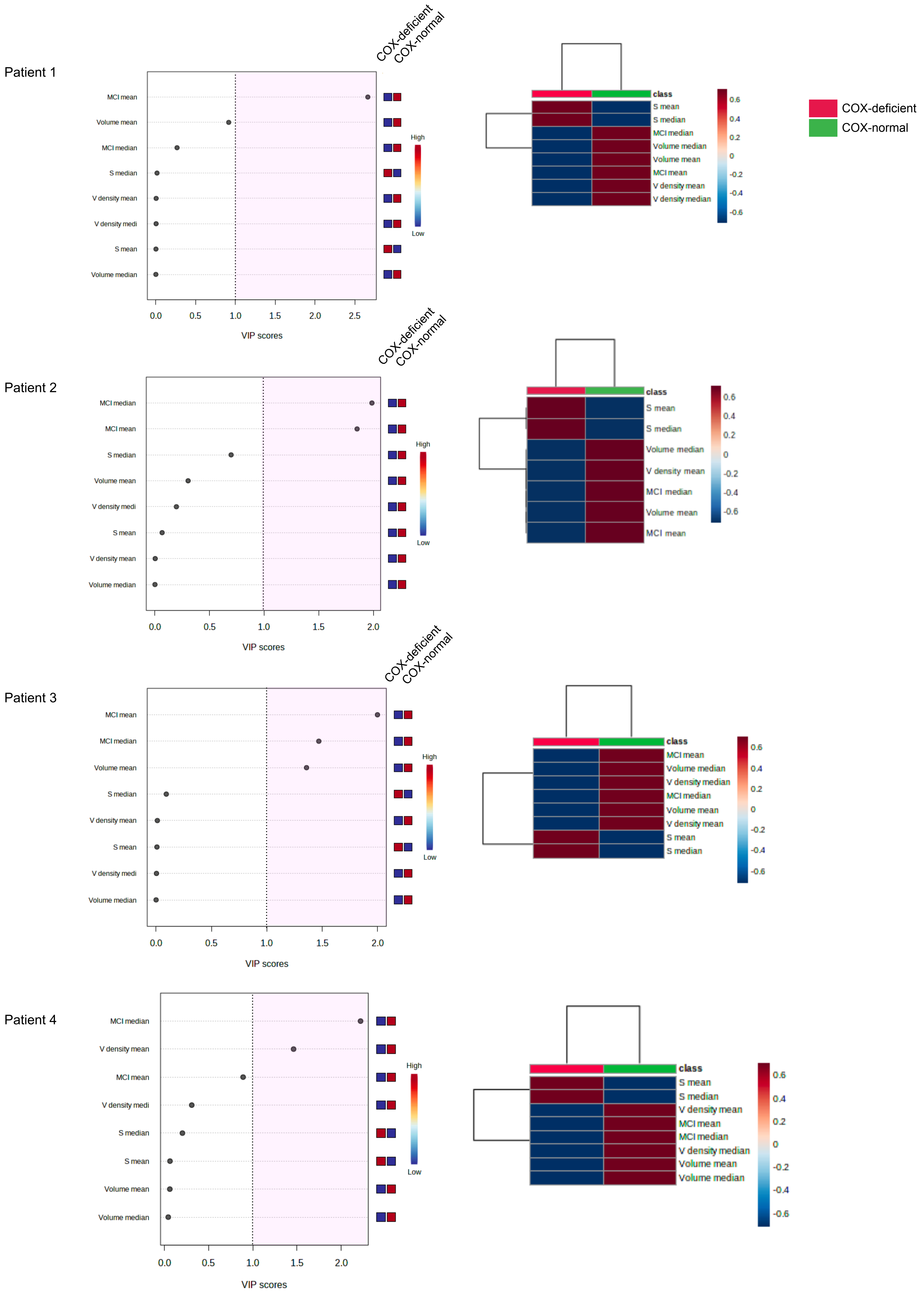

COX-deficient
COX-normal
Patient 1
MCI mean
Volume mean
MCI median
S median
V density mean
V density medi
S mean
Volume median
High
Low
0.0
0.5
1.0
1.5
2.0
2.5
VIP scores
class
S mean
S median
MCI median
Volume median
Volume mean
MCI mean
V density mean
V density median
0.6
0.4
0.2
0
-0.2
-0.4
-0.6
COX-deficient
COX-normal
Patient 2
MCI median
MCI mean
S median
Volume mean
V density medi
S mean
V density mean
Volume median
High
Low
0.0
0.5
1.0
1.5
2.0
VIP scores
class
S mean
S median
Volume median
V density mean
MCI median
Volume mean
MCI mean
0.6
0.4
0.2
0
-0.2
-0.4
-0.6
COX-deficient
COX-normal
Patient 3
MCI mean
MCI median
Volume mean
S median
V density mean
S mean
V density medi
Volume median
High
Low
0.0
0.5
1.0
1.5
2.0
VIP scores
class
MCI mean
Volume median
V density median
MCI median
Volume mean
V density mean
S mean
S median
0.6
0.4
0.2
0
-0.2
-0.4
-0.6
Patient 4
MCI median
V density mean
MCI mean
V density medi
S median
S mean
Volume mean
Volume median
High
Low
0.0
0.5
1.0
1.5
2.0
VIP scores
class
S mean
S median
V density mean
MCI mean
MCI median
V density median
Volume mean
Volume median
0.6
0.4
0.2
0
-0.2
-0.4
-0.6

Figure S2. Multivariate analysis of mitochondrial morphology between individual normal and deficient fibres from patient 1,2,3 & 4. Left panel: The variable importance in projection or VIP score for the morphology parameters used in the PLS-DA. The parameters with VIP score > 1 are considered significant. The coloured boxes on the right indicate the relative concentrations of the corresponding metabolite in each group under the current study for Patient 1,2,3 and 4. **Right panel:** Heatmap of group average with dendrograms illustrating hierarchical clustering of pattern similarity across morphological parameters and samples (top) (Euclidean distance measure, Ward clustering algorithm), for Patient 1,2,3 and 4. The colours indicate the relative quantitative value, where red indicates a higher value, and blue indicates a lower value.

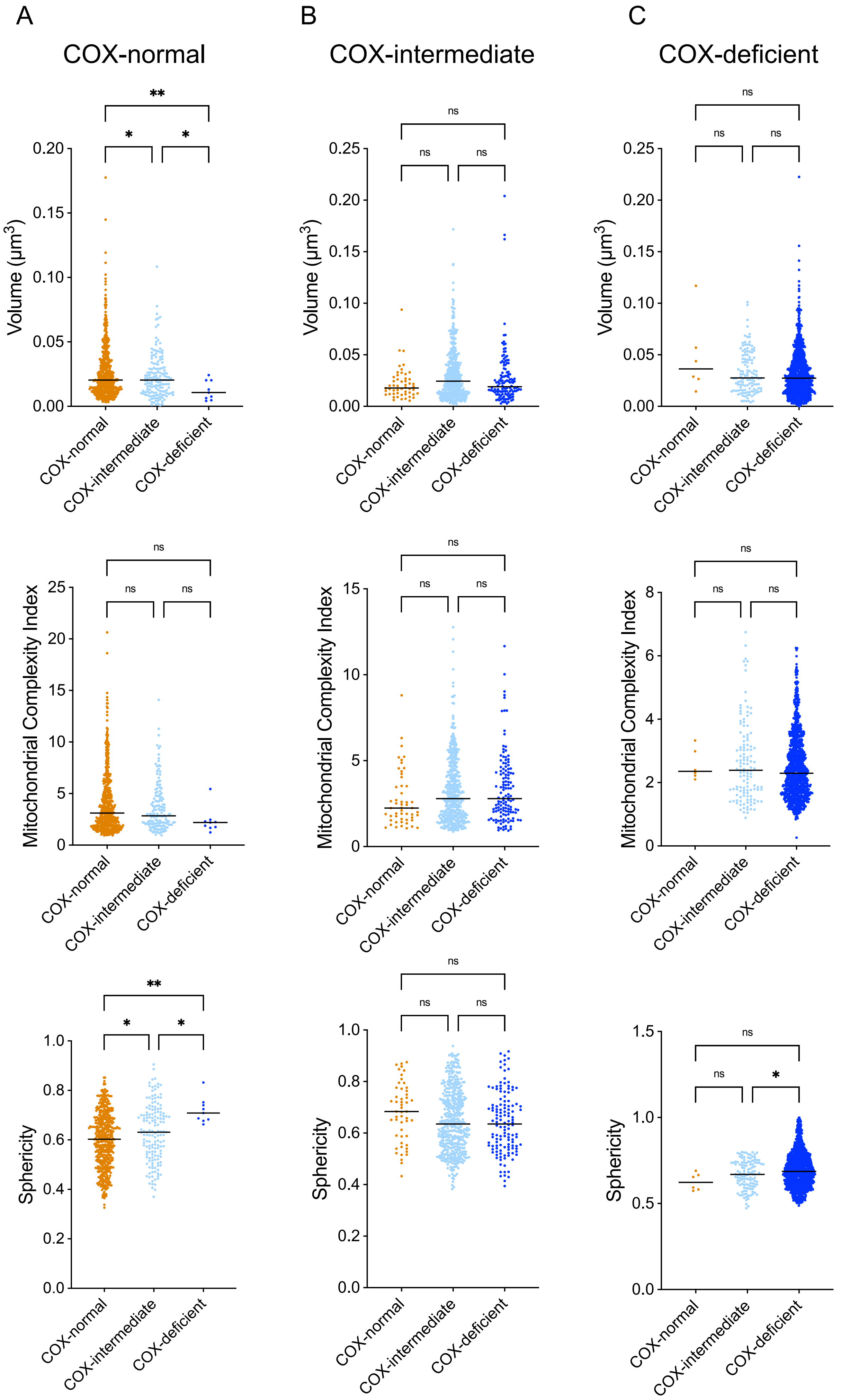

Figure S3. Mitochondrial morphology comparison from COX-normal, intermediate, and deficient fibres from Patient 1. Scatter plot showing mitochondrial volume **(A)**, MCI **(B)** and sphericity **(C)** of COX-normal, COX-intermediate and COX-deficient mitochondria from respective fiber.

COX normal fibre n=687 mitochondria, COX intermediate n= 637 mitochondria, COX deficient fibre n=1141 mitochondria

Data are presented as median with 95% CI. For multiple comparison (more than two groups) the Kruskal-Wallis test or Mann-Whitney (for two groups only) were applied, to examine the main effects, followed by posthoc tests using the two-stage step up method of Benjamini, Krieger, and Yekutieli to correct for multiple comparisons (*****$p < 0.05$, $q < 0.05$).

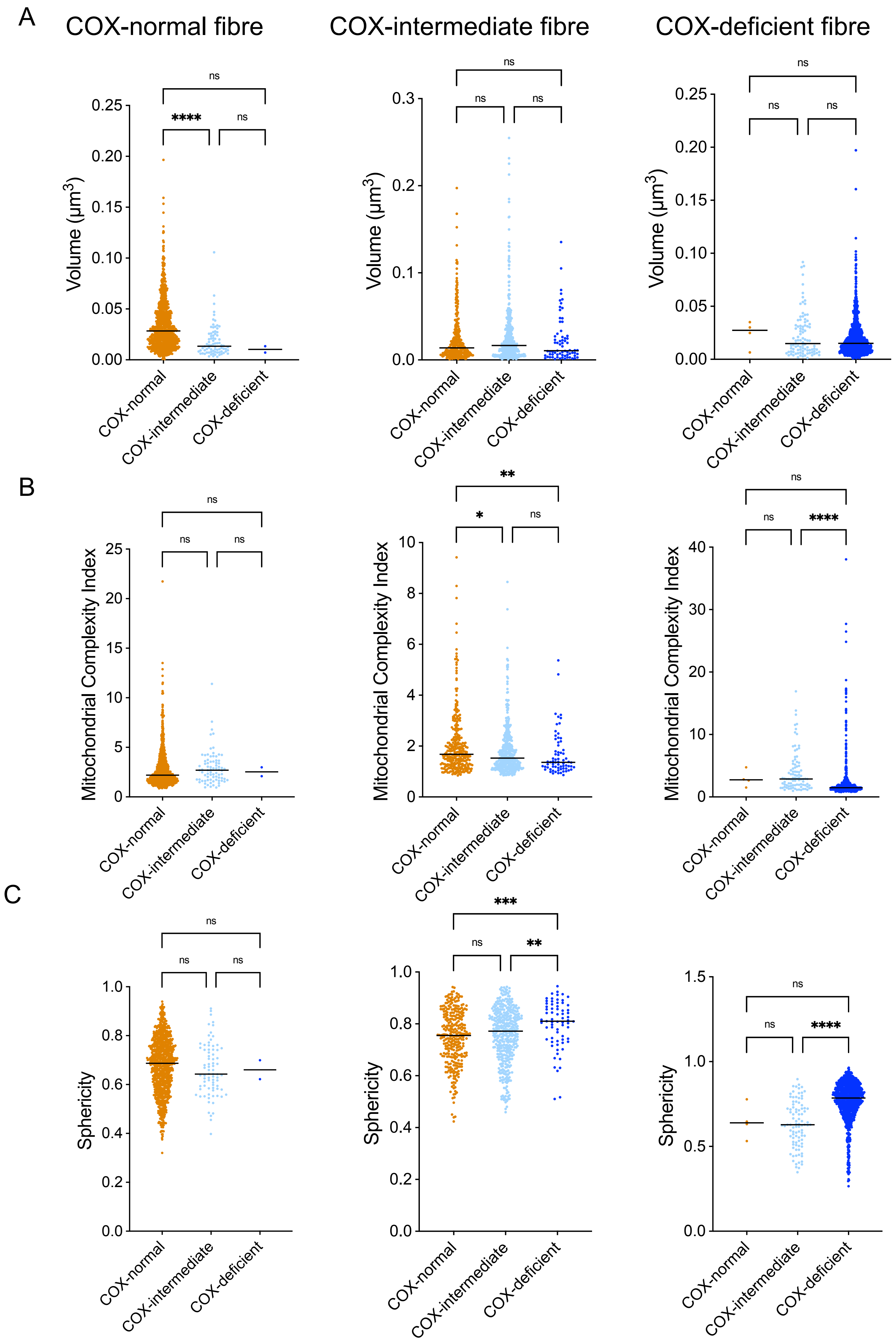

Figure S4. Mitochondrial morphology comparison from COX-normal, intermediate, and deficient fibres from Patient 2
Scatter plot showing mitochondrial volume **(A)**, MCI **(B)** and sphericity **(C)** of COX-normal, COX-intermediate and COX-deficient mitochondria from respective fiber.
COX normal fibre n=1043 mitochondria, COX intermediate n= 798 mitochondria, COX deficient fibre n=1102 mitochondria
Data are presented as median with 95% CI. For multiple comparison (more than two groups) the Kruskal-Wallis test or Mann-Whitney (for two groups only) were applied, to examine the main effects, followed by posthoc tests using the two-stage step up method of Benjamini, Krieger, and Yekutieli to correct for multiple comparisons (*****p < 0.05, q < 0.05).

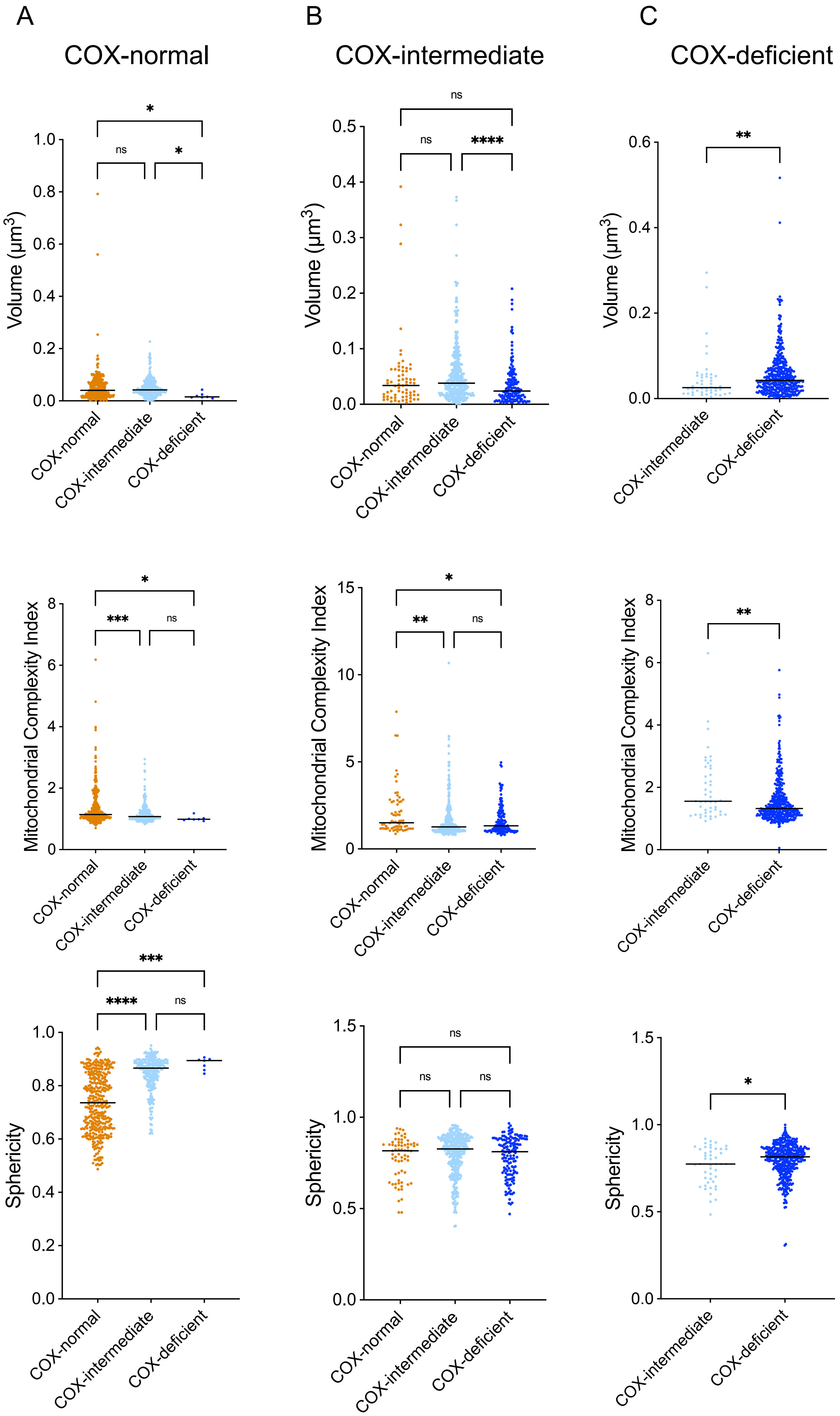

Figure S5. Mitochondrial morphology comparison from COX-normal, intermediate, and deficient fibres from Patient 3

Scatter plot showing mitochondrial volume **(A)**, MCI **(B)** and sphericity **(C)** of COX-normal, COX-intermediate and COX-deficient mitochondria from respective fiber.

COX normal fibre n=635 mitochondria, COX intermediate n= 548 mitochondria, COX deficient fibre n=462 mitochondria

Data are presented as median with 95% CI. For multiple comparison (more than two groups) the Kruskal-Wallis test or Mann-Whitney (for two groups only) were applied, to examine the main effects, followed by posthoc tests using the two-stage step up method of Benjamini, Krieger, and Yekutieli to correct for multiple comparisons (*****$p < 0.05$, $q < 0.05$).

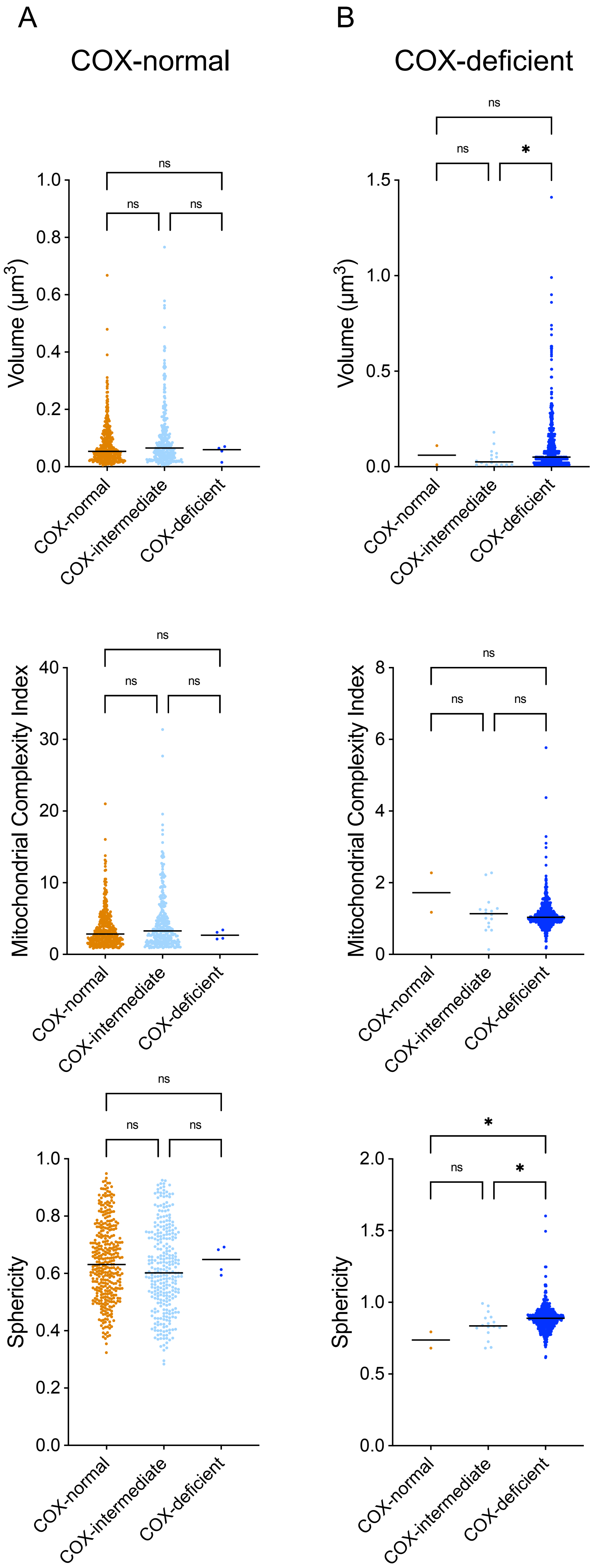

Figure S6. Mitochondrial morphology comparison from COX-normal, intermediate, and deficient fibres from Patient 4
Scatter plot showing mitochondrial volume **(A)**, MCI **(B)** and sphericity **(C)** of COX-normal, COX-intermediate and COX-deficient mitochondria from respective fiber. COX normal fibre n=659 mitochondria, COX deficient fibre n=606 mitochondria. Data are presented as median with 95% CI. For multiple comparison (more than two groups) the Kruskal-Wallis test or Mann-Whitney (for two groups only) were applied, to examine the main effects, followed by posthoc tests using the two-stage step up method of Benjamini, Krieger, and Yekutieli to correct for multiple comparisons (*****$p < 0.05$, $q < 0.05$).

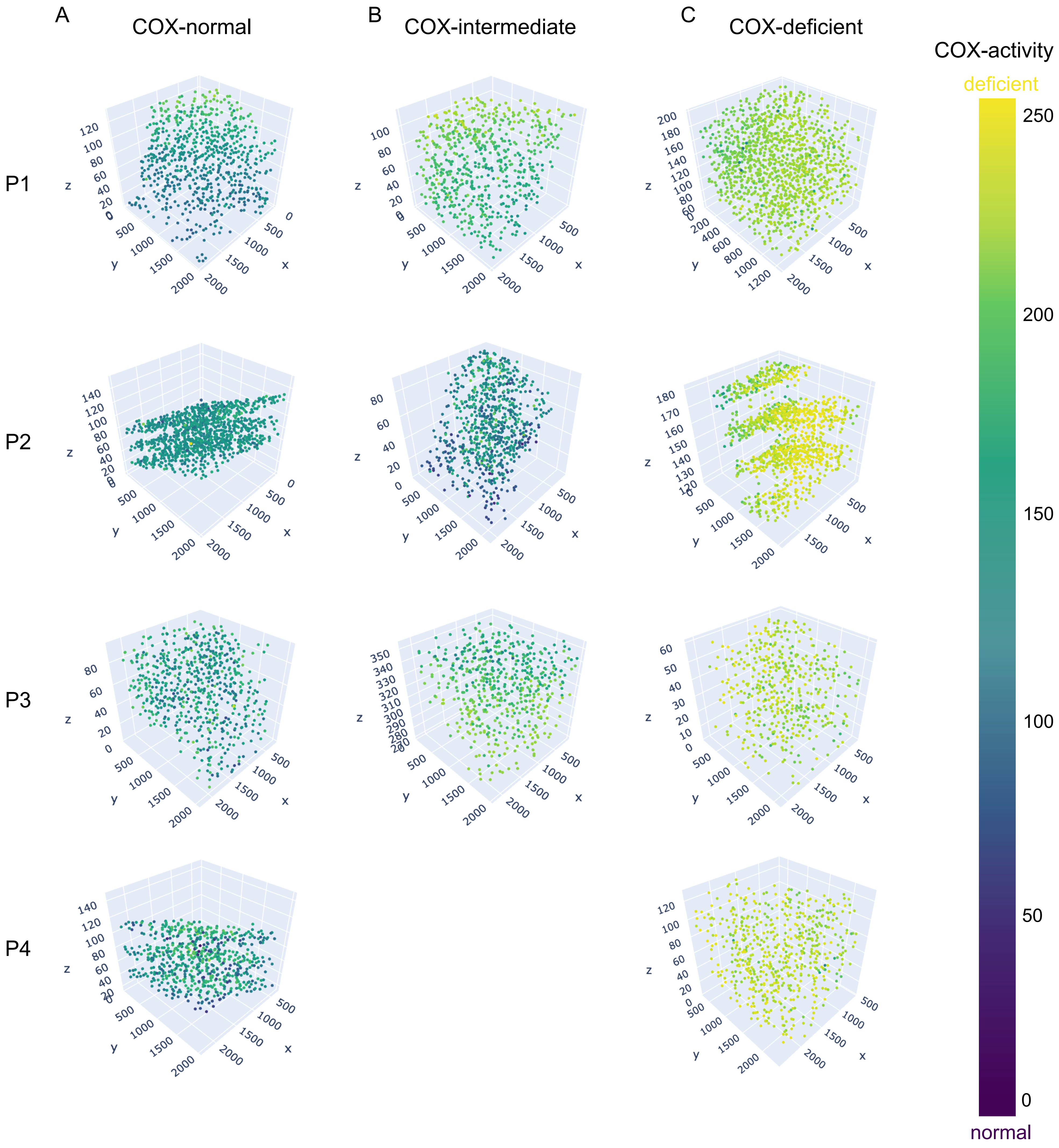

Figure S7. COX activity and spatial distribution of the mitochondrial COX activity and their respective morphologies within a COX-normal/COX-intermediate and COX-deficient fibres from Patient 1,2,3&4 from figure 8

The 3D scatter plot of a COX-normal (Figure 7A), COX-intermediate (Figure 7B) and COX-deficient (Figure 7C) fibre demonstrate coloured mitochondria according to their COX activity.

3D scatter plot of mitochondrial COX activity across two sarcomeres in COX normal **(A),** COX-intermediate **(B)** and COX-deficient **(C)** fibres demonstrate coloured mitochondria according to their COX activity for Patient 1,2,3 and 4.

Mitochondria are colour coded dependent on COX activity as indicated by the scale Cluster of COX-normal and intermediate mitochondria can be distinguished by the dotted circle.

Patients	Fibre ID	COX normal (%)	COX intermediate (%)	COX deficient (%)
Patient1	Fibre 1	82.3	17.4	0.3
	Fibre 8	71.6	27.9	0.4
	Fibre 13	92.5	7.5	0
	Fibre 16	62.3	37.7	0
	Fibre 14	87.8	12.2	0
	Fibre 2	8.8	88.8	2.4
	Fibre 9	7.5	91.9	0.6
	Fibre 10	5.5	79.6	14.9
	Fibre 15	13.9	85	1.1
	Fibre 3	0.5	22.1	77.4
	Fibre 4	0.1	10.5	89.4
	Fibre 5	0.1	6.6	93.3
	Fibre 6	0	0.8	99.2
	Fibre 7	0	14.2	85.8
	Fibre 11	0	41.7	58.3
	Fibre 12	0	41.7	58.3
Patient 2	Fibre 1	54.5	44.6	0.8
	Fibre 5	69.4	29.4	1.2
	Fibre 6	85.6	14.3	0.1
	Fibre 7	74.2	25.8	0
	Fibre 12	84	15.9	0.1
	Fibre 2	15.8	80.2	4
	Fibre 4	24.3	74.3	1.5
	Fibre 11	34.5	61.7	3.9
	Fibre 3	0.1	18.2	81.7
	Fibre 8	0.5	29.8	69.7
	Fibre 9	0.2	18.2	81.6
	Fibre 10	0.4	11.6	88
	Fibre 13	0.2	5.2	94.7
Fibre 3	Fibre 4	60.32	36.22	3.46
	Fibre 6	54.78	43.1	2.12
	Fibre 7	77.8	21.8	0.4
	Fibre 3	12.23	60.95	26.82
	Fibre 1	0	9.95	90.05
	Fibre 2	0.42	23.22	76.36
	Fibre 5	0.26	16.82	82.92
Fibre 4	Fibre 6	56.80	45.2	2
	Fibre 7	75.2	23.3	1.4
	Fibre 1	6.59	26.44	66.97
	Fibre 2	0.25	2.4	97.35
	Fibre 5	0	0.8	99.2
	Fibre 4	0.33	1.65	98.02
	Fibre 3	0.44	3.65	95.91

Table S1 Percentage class of individual mitochondria in fibre of patients P1,P2,P3 & P4.

	P1			P2			P3			P4	
	F+	F±	F-	F+	F±	F-	F+	F±	F-	F+	F-
V < 10%	10,15	20,58	30,91	10,05	14,19	26,44	10,05	15,15	15,47	9,91	11,17
10% < V < 90%	79,87	78,51	68,54	79,98	71,75	69,43	79,97	84,85	84,12	80,18	83,29
V > 90%	9,97	0,91	0,55	9,97	14,06	4,13	9,98	0,00	0,41	9,91	5,54
MCI < 10%	10,03	30,86	43,51	10,01	13,74	31,29	9,98	5,66	14,49	9,91	66,39
10% < MCI < 90%	79,99	67,58	55,35	80,00	79,33	65,85	80,04	94,16	85,22	80,18	33,56
MCI > 90%	9,97	1,56	1,13	9,99	6,93	2,85	9,98	0,18	0,29	9,91	0,06
S < 10%	10,00	1,56	1,13	9,97	6,93	2,85	9,98	0,18	0,29	9,91	0,19
10% < S < 90%	80,02	67,59	55,41	80,09	79,35	65,87	80,11	94,16	85,22	80,18	44,38
S > 90%	9,97	30,85	43,45	9,95	13,72	31,28	9,91	5,66	14,49	9,91	55,43

Table S2. Proportion of simple and complex mitochondria of COX-normal, intermediate and deficient fibres from all patients.

Table represent the 10th and 90th percentiles for volume, MCI and sphericity of mitochondria from COX-normal. Small and simple mitochondria with volume and MCI values inferior to the 10th percentile of mitochondria from COX-normal fibres. Large and complex mitochondria are those with volume and MCI values superior to the 90th percentile of mitochondria from COX-normal. Spherical mitochondria are those with values superior to the 90th percentile of mitochondria from COX-normal.
